# Second-generation bone cement-injectable cannulated pedicle screws for osteoporosis: biomechanical and finite element analyses

**DOI:** 10.1186/s13018-023-03752-2

**Published:** 2023-05-10

**Authors:** Congcan Li, Lei Song, Jun Xiao, Wenwen Wu, Yifan Jiang, Rui Zhou, Fei Dai

**Affiliations:** 1 Department of Orthopaedics, First Affiliated Hospital, Army Medical University, No. 30 Gaotanyanzheng Street, Chongqing, 400038 China; 2 Department of Special Service Physiological Training, Guangzhou Special Service Recuperation Center of PLA Rocket Force, Shantou, 515515 China; 3 Chinese People’s Liberation Army 132U, Tunchang, 571627 China; 4 Fourth Department of Convalescence, Sanya Rehabilitation and Convalescent Center, Joint Logistics Support Force, Sanya, 572000 China

**Keywords:** Cement-injectable cannulated pedicle screws, Osteoporosis, Safety, Stability

## Abstract

**Background:**

Biomechanical and finite element analyses were performed to investigate the efficacy of second-generation bone cement-injectable cannulated pedicle screws (CICPS) in osteoporosis.

**Methods:**

This study used the biomechanical test module of polyurethane to simulate osteoporotic cancellous bone. Polymethylmethacrylate (PMMA) bone cement was used to anchor the pedicle screws in the module. The specimens were divided into two groups for the mechanical tests: the experimental group (second-generation CICPS) and control group (first-generation CICPS). Safety was evaluated using maximum shear force, static bending, and dynamic bending tests. Biomechanical stability evaluations included the maximum axial pullout force and rotary torque tests. X-ray imaging and computed tomography were used to evaluate the distribution of bone cement 24 h after PMMA injection, and stress distribution at the screw fracture and screw–cement–bone interface was assessed using finite element analysis.

**Results:**

Mechanical testing revealed that the experimental group (349.8 ± 28.6 N) had a higher maximum axial pullout force than the control group (277.3 ± 8.6 N; *P* < 0.05). The bending moments of the experimental group (128.5 ± 9.08 N) were comparable to those of the control group (113.4 ± 20.9 N; *P* > 0.05). The screw-in and spin-out torques of the experimental group were higher than those of the control group (spin-in, 0.793 ± 0.015 vs. 0.577 ± 0.062 N, *P* < 0.01; spin-out, 0.764 ± 0.027 vs. 0.612 ± 0.049 N, *P* < 0.01). Bone cement was mainly distributed at the front three-fifths of the screw in both groups, but the distribution was more uniform in the experimental group than in the control group. After pullout, the bone cement was closely connected to the screw, without loosening or fragmentation. In the finite element analysis, stress on the second-generation CICPS was concentrated at the proximal screw outlet, whereas stress on the first-generation CICPS was concentrated at the screw neck, and the screw–bone cement–bone interface stress of the experimental group was smaller than that of the control group.

**Conclusion:**

These findings suggest that second-generation CICPS have higher safety and stability than first-generation CICPS and may be a superior choice for the treatment of osteoporosis.

## Introduction

An aging population is increasingly becoming a global problem, and spinal degenerative diseases are now frequently diagnosed in older patients. Pain caused by intervertebral disc herniation, spinal stenosis, and spondylolysis impose a heavy burden on the economy, and seriously affect the health and quality of life of patients [1]. Surgery is indicated when conservative treatment fails to achieve spinal recovery.

Pedicle screw fixation has been widely used in the surgical treatment of spinal degenerative diseases [2–5]; however, patients with osteoporosis provide a greater challenge. Osteoporosis seriously affects bonding at the bone–screw interface and screw stability owing to the destruction of bone tissue microstructure and reduction of bone mass; this results in screw loosening, displacement, or protrusion [6, 7].

Polymethylmethacrylate (PMMA)-reinforced screws have been proven to be the most effective system for strengthening pedicle screws and achieving stable fixation [2, 8–11]. PMMA bone cement penetrates the bone trabeculae to form a cement–bone interface in addition to the screw–bone interface, improving the stability of pedicle screws in osteoporotic vertebrae [12–14].

Since the initial application of first-generation cement-injectable cannulated pedicle screws (CICPS) enhanced with PMMA in clinical practice in 2011, the incidence of screw loosening, bone cement leakage, and even pulmonary embolism has reduced [15–18].

In 1975, Liu et al. [19] constructed the first three-dimensional finite element model of the vertebral body for the study of direct shear resistance in the lumbar spine, marking the beginning of finite element analysis in spinal biomechanics. This analysis has been increasingly used with the rapid development of computer technology [20, 21] and involves the creation of a detailed and accurate computer-simulated three-dimensional stereo model [22, 23]. Stress analysis can then be performed using this model, which greatly assists in the correct selection of surgical methods, reducing the rates of surgical failure and complications, and optimizing implant design.

To further optimize screw design and reduce complications, we have improved the design of first-generation CICPS to create second-generation CICPS. This study aimed to evaluate screw safety through biomechanical experiments and analyze stress distribution at the screw fracture and screw–cement–bone interface using finite element analysis.

## Methods

### Screw design

**Fig. 1:**
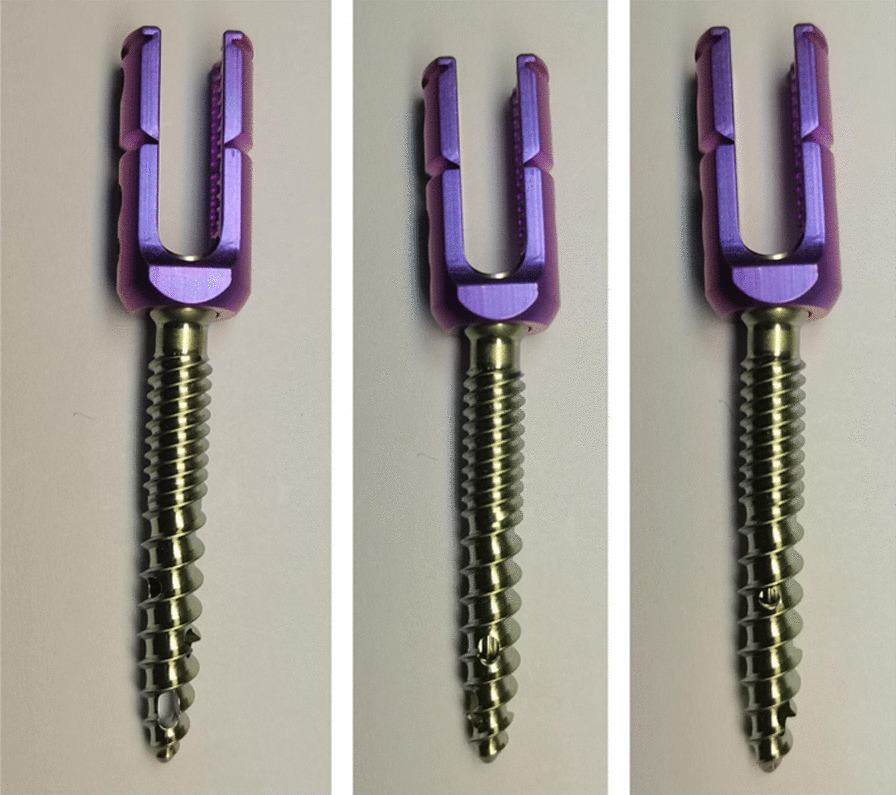
The proximal, middle, and distal side holes are shown in the left, middle, and right panels, respectively

Second-generation CICPS design features a dual thread with an inner diameter of 1.6 mm, a thread lead of 6 mm, and a pitch of 1.5 mm in the proximal two-fifths and 3 mm in the distal three-fifths. The three side holes in the front two-fifths of the screw are separated from each other by 120° and two threads. From the near to far sides, the holes are round (2 × 2 mm), oval (3 × 2 mm), and U-shaped (4 × 2 mm) and can be designed with different specifications. All pedicle screws used in this study were manufactured by Chongqing FWS Medical Device Co., Ltd. (Chongqing, China) and had a diameter of 6.5 mm and a length of 45 mm (Fig. 1).

### Specimen preparation

**Fig. 2:**
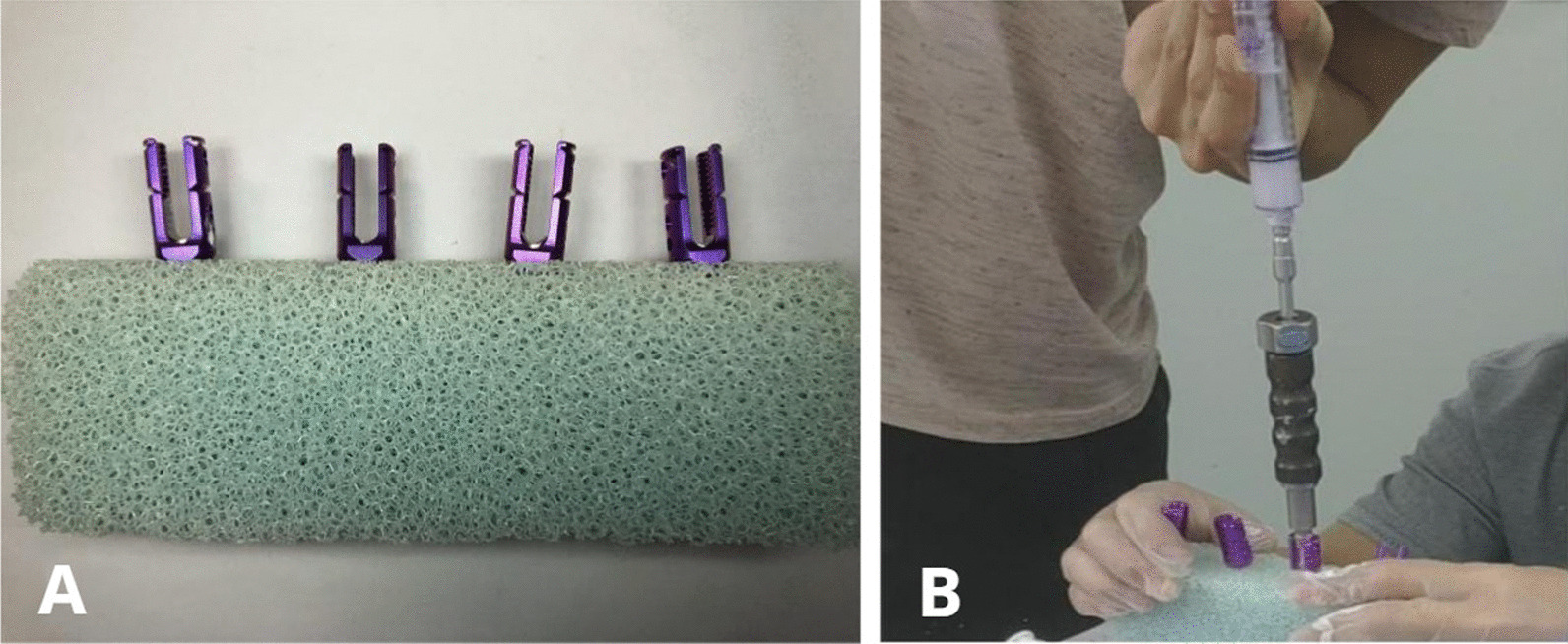
Screw placement was done according to the standard clinical method (**A**) and was followed by injection of 1.5 mL PMMA into each screw at room temperature (**B**)

Commercially available polyurethane (PU; catalog no. 1522-507; open cell 7.5 PCF, 18 × 13 × 4 cm; Sawbones, Pacific Research Laboratories, Vashon Island, WA, USA) was used to simulate the mechanical properties of osteoporotic cancellous bone. An open cone was used to drill holes in the side of the specimen, and a nail hole with a depth of 40 mm was prepared perpendicular to the side of the specimen. After installation of the screw placement instrument, the pedicle screw was slowly screwed vertically into the cancellous bone substitute, until the screw head was in contact with the surface of the specimen. During screw placement, the specimen structure was protected from damage by left–right shaking. All specimens were used self-tapping screws to avoid weakening the screw-holding force (Fig. 2A).

### Bone cement injection

At room temperature, 20 g PMMA bone cement and 10 mL solvent were mixed in a stainless-steel bowl, with a strict powder to liquid ratio of 2:1. When the bone cement became a paste, 10 mL was placed into a syringe, and the air was removed from the connecting device and screw. When the bone cement had developed the consistency of toothpaste, 1.5 mL was injected into each screw (Fig. 2B). After allowing the bone cement to set for 24 h, X-ray imaging and computed tomography (CT) were performed to observe the distribution of the bone cement in the module.

### Mechanical testing

#### Static bending test

**Fig. 3:**
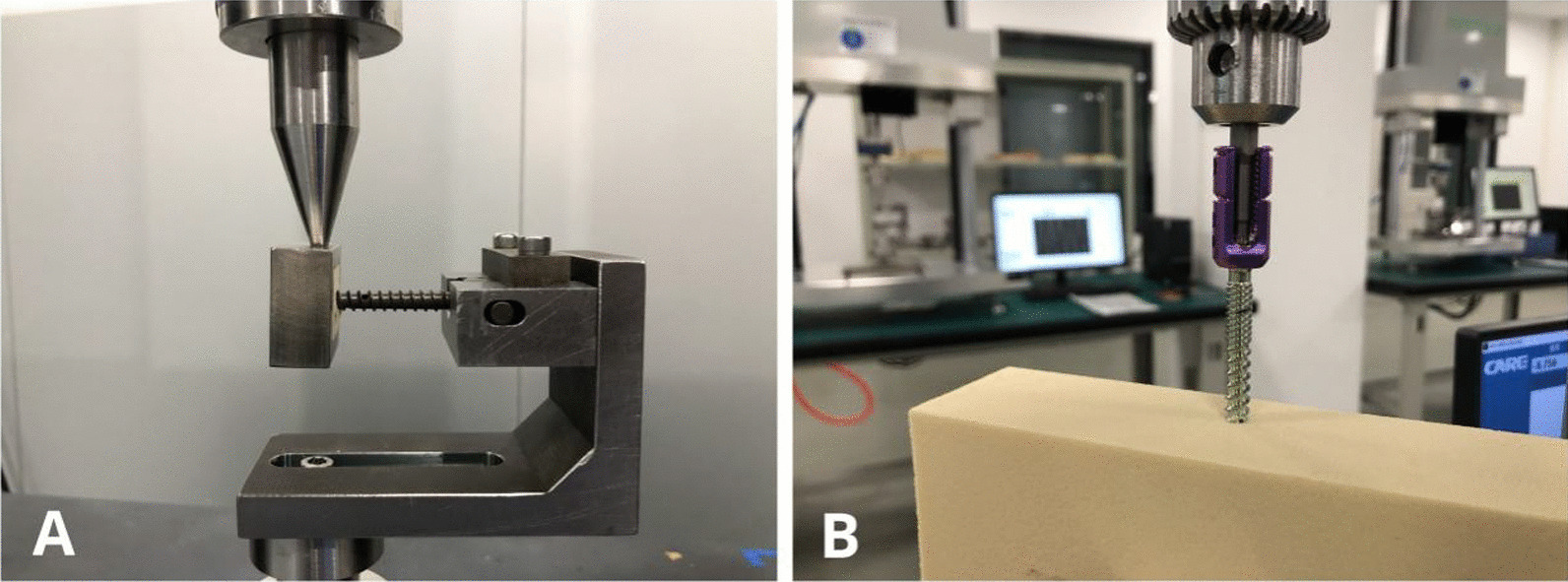
Experimental setups for the static bending test (**A**) and rotary torque test (**B**)

This was performed using an electronic universal testing machine (model no. CMT-5105). The long axis of the adjusting screw was perpendicular to the bending direction, and the load was applied at a constant rate of 25 mm/min perpendicular to the long axis of the pedicle screw. A computer automatically recorded the continuity value and drew the load–displacement curve until the load dropped significantly (Fig. 3A).

#### Dynamic bending test

This was performed using a fatigue tester (model no. Fd3000-P), with the screw installed in the same manner as in the above test, and the maximum loads set at 65%, 75%, and 80% of the yield load. The loading wave was sinusoidal, R-value was 0.1, and frequency was 10 Hz. Dynamic tests were conducted for each screw in turn, and the load–cycle curves were drawn using a computer analysis of the results until the screw broke or the cycle stopped at 2.5 × 10^6^ cycles.

#### Rotary torque test

This was performed using a surgical implant electromagnetic dynamic mechanics test system (model no. M-100T). The adjusting screw was perpendicular to the specimen surface and a force of 11.17 N was applied vertically at a constant torsional speed of 3 r/min. The computer automatically recorded the continuity value and drew the torque–angular displacement curve; the recording stopped when all threads had entered the module. Unscrewing was performed in the same manner until the threads had completely exited the module (Fig. 3B).

#### Maximum axial pullout force test

**Fig. 4:**
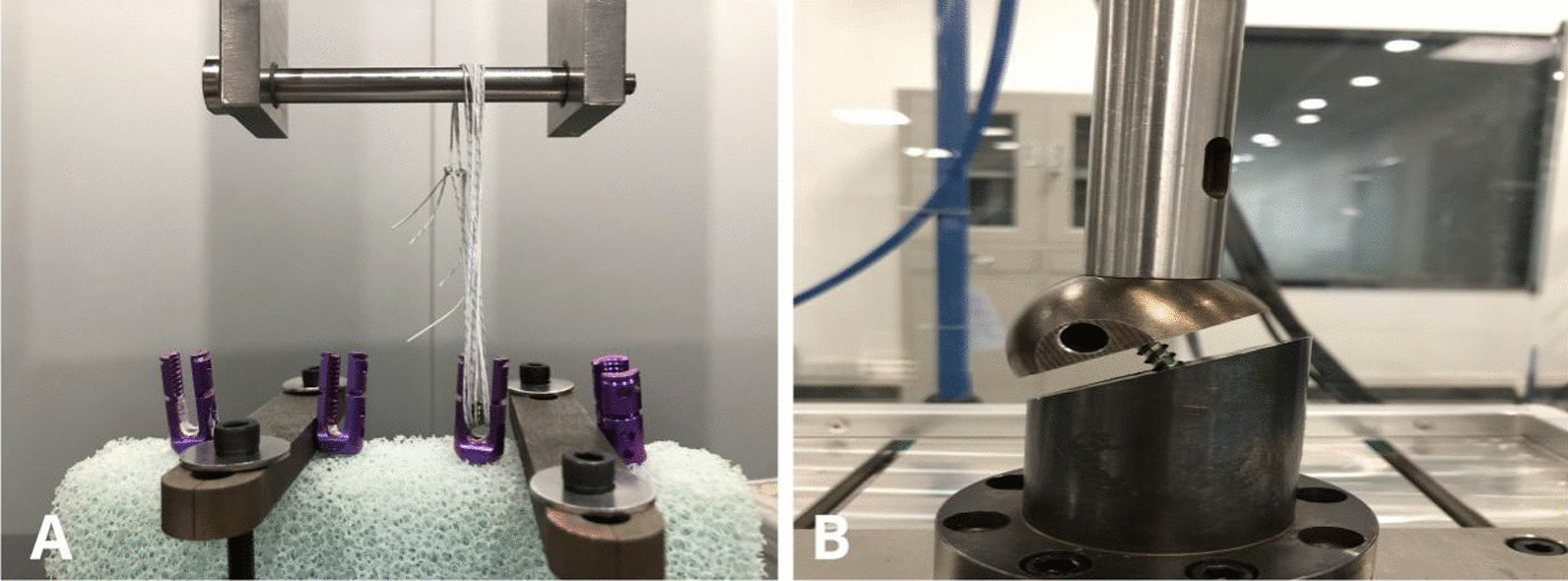
Experimental setups for the maximum axial pullout force test (**A**) and maximum shear force test (**B**)

This was performed using an electronic universal testing machine (model no. CMT-5105). The long axis of the adjusting screw was parallel to the stretching direction. The screw was pulled out along the long axis of the pedicle screw at a constant rate of 5 mm/min. The computer automatically recorded the continuity value and drew the load–displacement curve until the load dropped significantly (Fig. 4A).

#### Maximum shear force test

The test element was a fatigue tester (model no. FD5000-P) with maximum loads of 1000, 1500, and 2000 N. The loading wave was a sine wave with an R-value of 0.1 and a frequency of 5 Hz. Dynamic tests were carried out for each screw in turn, and the load–cycle curves were drawn by computer analysis of the results until the screw broke or the cycles stopped at 2.5 × 10^6^ cycles (Fig. 4B).

### Finite element analyses

**Fig. 5:**
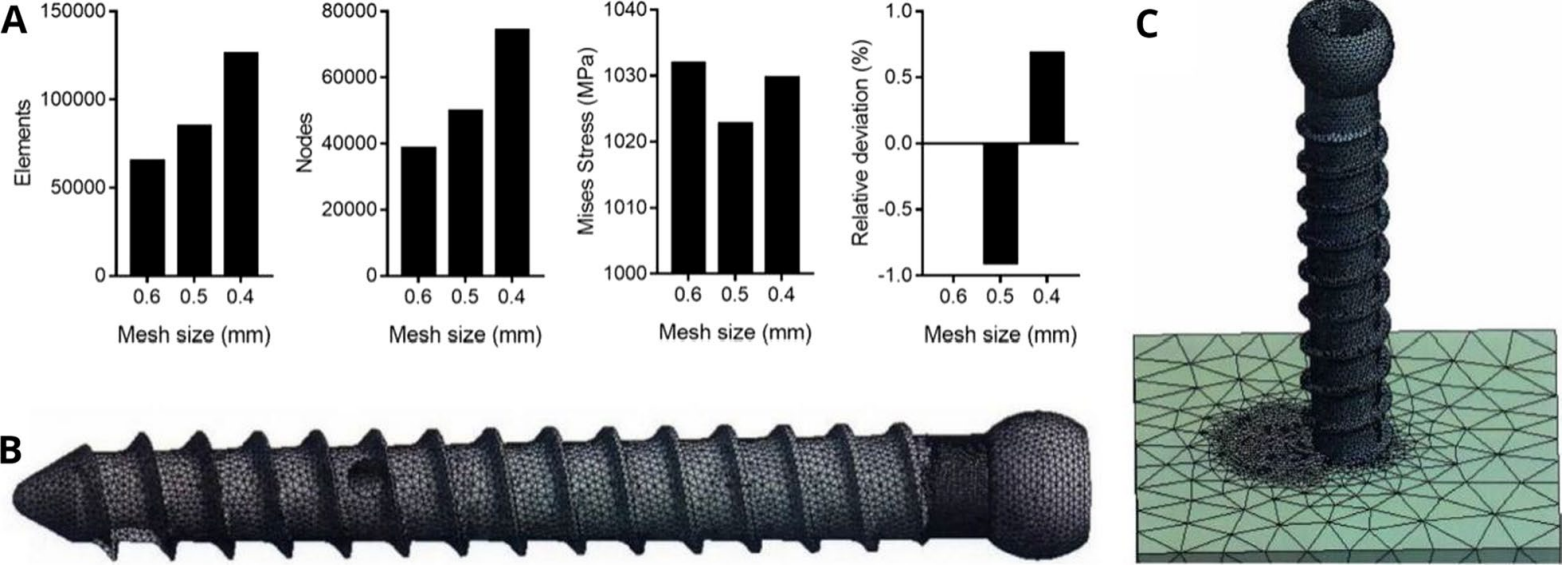
Convergence analysis results of the finite element mesh (**A**), screw model (**B**), and screw–bone cement–bone model (**C**)

The CT scan results at 0.625 mm intervals were imported into SolidWorks geometric model processing software version 1.0 (SolidWorks Corp., Waltham, MA, USA), and ANSYS Workbench software version 19.0 (Ansys, Inc., Canonsburg, PA, USA) was used for the finite element calculation. Screw and screw–cement–bone models were established by meshing. A grid convergence analysis was conducted, and the network type was a 10-node tetrahedral element, as shown in Fig. 5A. The results indicated that the maximum stress difference was less than 5% and that the model converged. Considering the calculation accuracy, speed, and stress distribution of the key parts, a 0.04 mm element size was adopted for mesh division in all calculations, and 0.04 mm was also used for all contact surface elements [24, 25].

Screw (Fig. 5B) and screw–bone cement–bone models (Fig. 5C) were established according to the elastic modulus and Poisson’s ratio of each material in the model [26, 27]. The screw model is a homogeneous, continuous, isotropic, and linearly elastic material [17, 18]. Bone is also considered to be homogeneous, isotropic, and linearly elastic [28]. In the screw–bone cement–bone model, friction contact exists between the screw, bone cement, and bone block [18, 29]. The maximum shear force test was used to evaluate the stress distribution at the screw fracture, and the maximum axial pullout test was used to evaluate the stress distribution at the screw–cement–bone interface.

Table 1 lists the mechanical properties of the investigated material [17, 29].

**Table 1:** Properties of materials used in this study

Material	*E* (Young’s modulus)	*v* (Poisson’s ratio)
Pedicle screw (Ti alloy)	110 GPa	0.3
Polymethylmethacrylate (PMMA)	2.27 GPa	0.46
Cancellous bone model (rigid polyurethane foam)	320 MPa	0.25

### Statistical analysis

All statistical analyses were performed using SPSS software version 21 (IBM Corp., Armonk, NY, USA); a *P* value < 0.05 was considered significant. Comparisons of all indices between the two groups were performed using a two-tailed Student’s t-test or analysis of variance.

## Results

### Mechanical testing

#### Static bending test

**Fig. 6:**
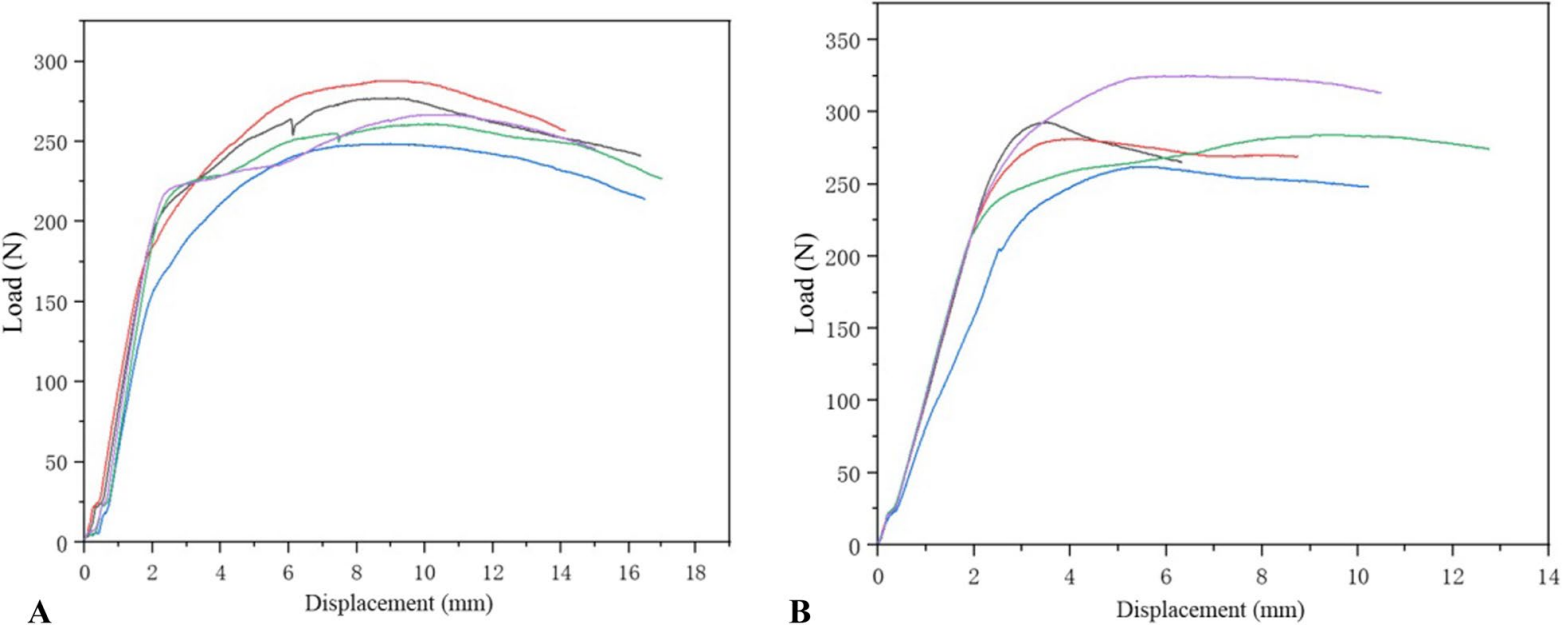
Load–displacement curves of second-generation (**A**) and first-generation (**B**) cement-injectable cannulated pedicle screws

As shown in Fig. 6, no significant differences were noted in bending stiffness (128.5 ± 9.08 vs. 113.4 ± 20.9 N), bending structure stiffness (3.13 ± 0.22 vs. 2.76 ± 0.508 N), and ultimate bending moment (268.9 ± 15.1 vs. 289.0 ± 23.0 N) between the two generations of screws, indicating that the two generations of CICPS had similar bending moments (*P* > 0.05).

#### Dynamic bending test

**Fig. 7:**
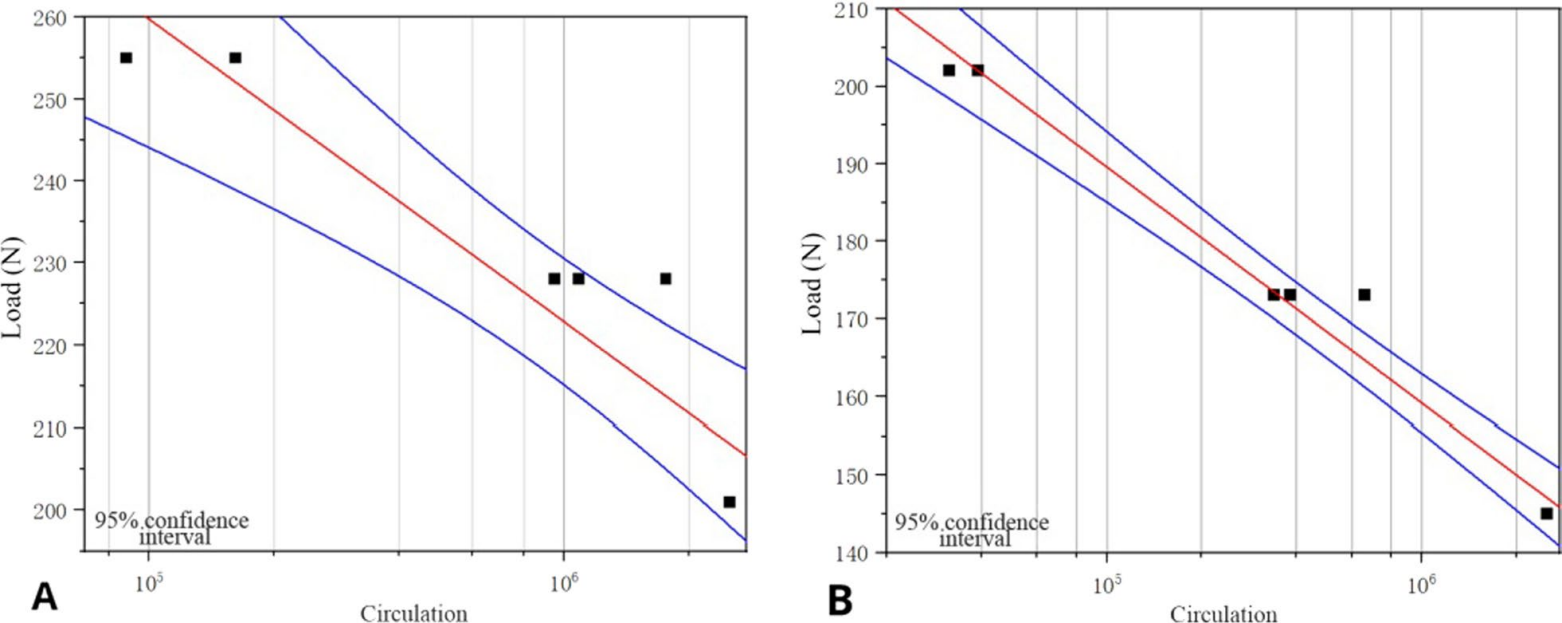
Load–cycle curves of second-generation (**A**) and first-generation (**B**) cement-injectable cannulated pedicle screws

**Fig. 8:**
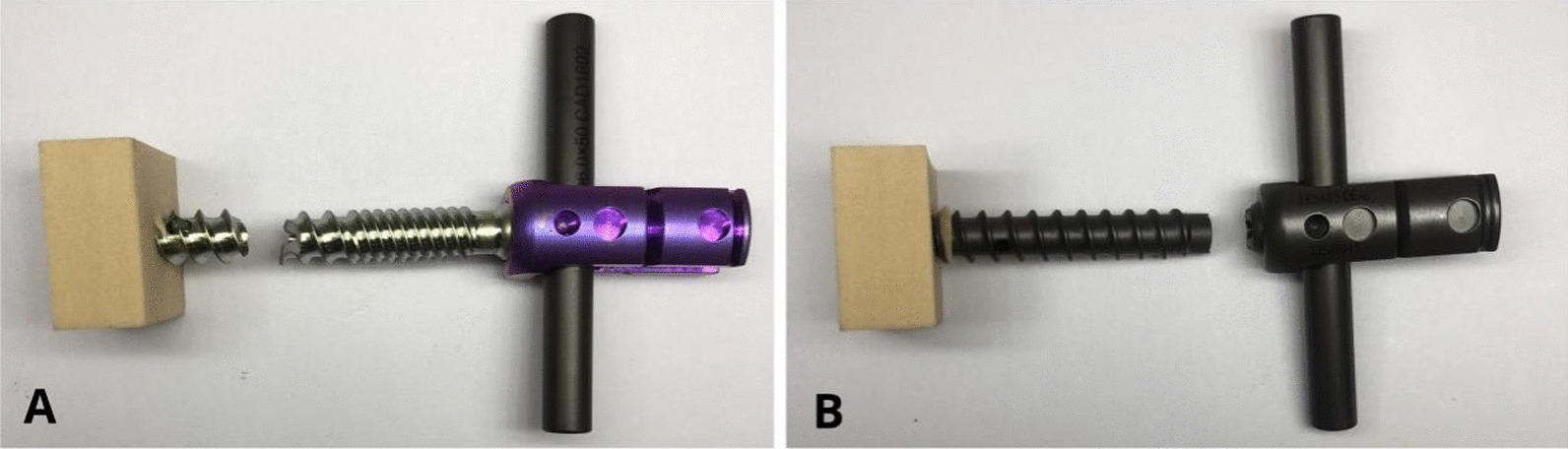
Second-generation screw fracture (**A**) and first-generation screw fracture (**B**)

As presented in Fig. 7, the second-generation CICPS passed 75% of the test of its ultimate bending moment, with fracture at the proximal side hole in 85% and 95% of the tests (Fig. 8A). The first-generation CICPS passed 50% of the test of its ultimate bending moment, and the screw neck broke in 60% and 70% of the tests (Fig. 8B). Second-generation CICPS can, therefore, withstand higher loads and more cycles before failure.

#### Rotary torque test

**Fig. 9:**
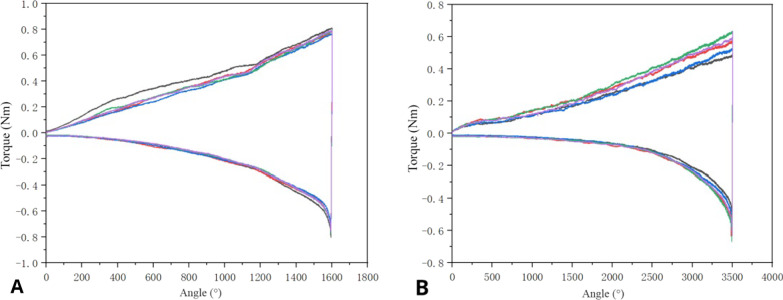
Torque–angular displacement curves of second-generation (**A**) and first-generation (**B**) cement-injectable cannulated pedicle screws

As presented in Fig. 9, the torque of the second-generation CICPS (0.793 ± 0.015 N) was higher than that of the first-generation CICPS (0.577 ± 0.062 N; *P* < 0.01). The spin-out torque of second-generation CICPS (0.764 ± 0.027 N) was also higher than that of first-generation CICPS (0.612 ± 0.049 N; *P* < 0.01).

#### Maximum axial pullout force test

**Fig. 10:**
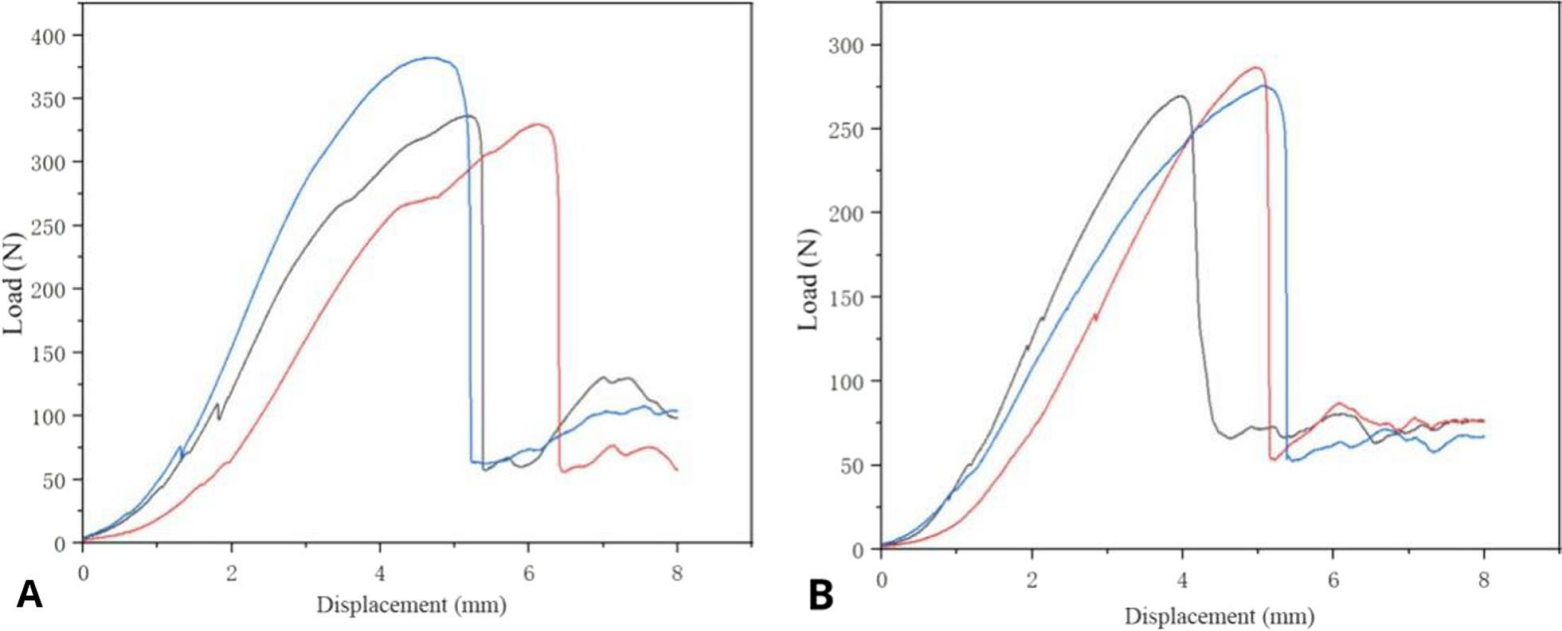
Load–displacement curves of second-generation (**A**) and first-generation (**B**) cement-injectable cannulated pedicle screws

As shown in Fig. 10, the maximum axial pullout force of second-generation CICPS (349.8 ± 28.6 N) was higher than that of first-generation CICPS (277.3 ± 8.6 N; *P* < 0.05).

#### Maximum shear force test

**Fig. 11:**
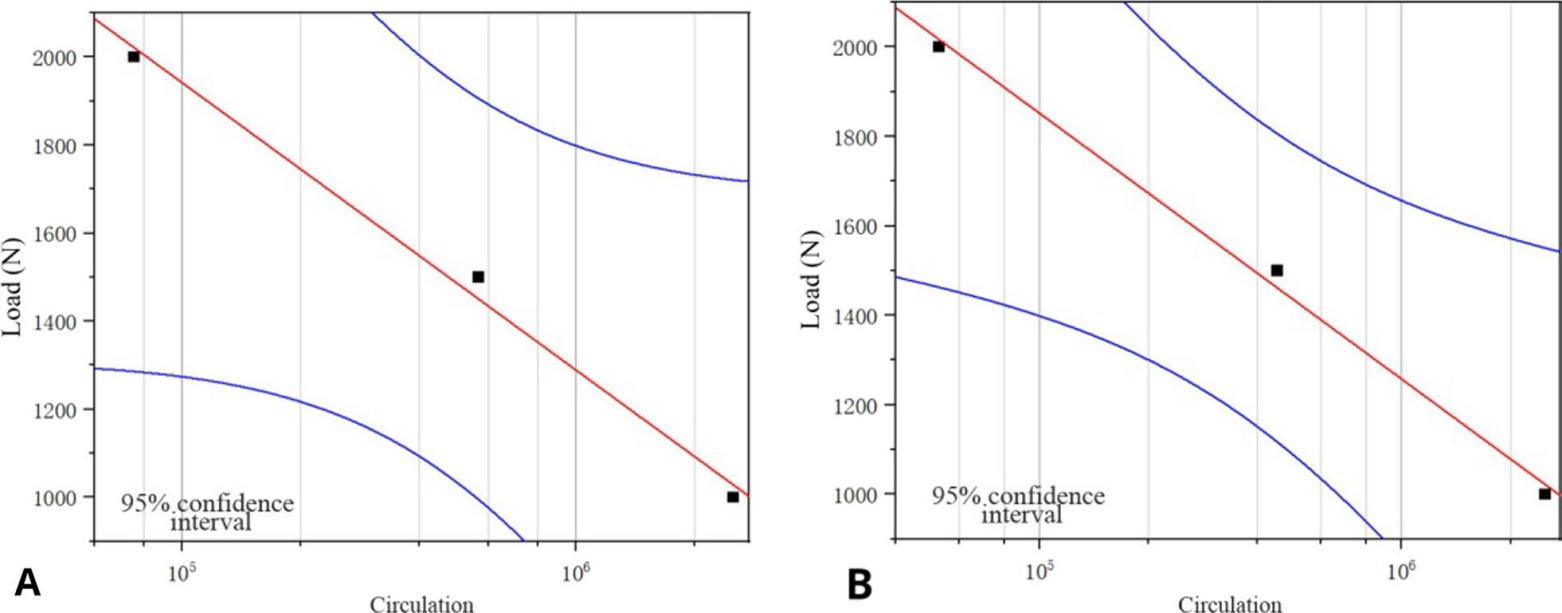
Load–cycle curves of second-generation (**A**) and first-generation (**B**) cement-injectable cannulated pedicle screws

As presented in Fig. 11, the two generations of screws passed the 1000 N load test and broke in the 1500 and 2000 N tests; however, the second-generation CICPS was able to withstand more cycles.

### Finite element analyses

**Fig. 12:**
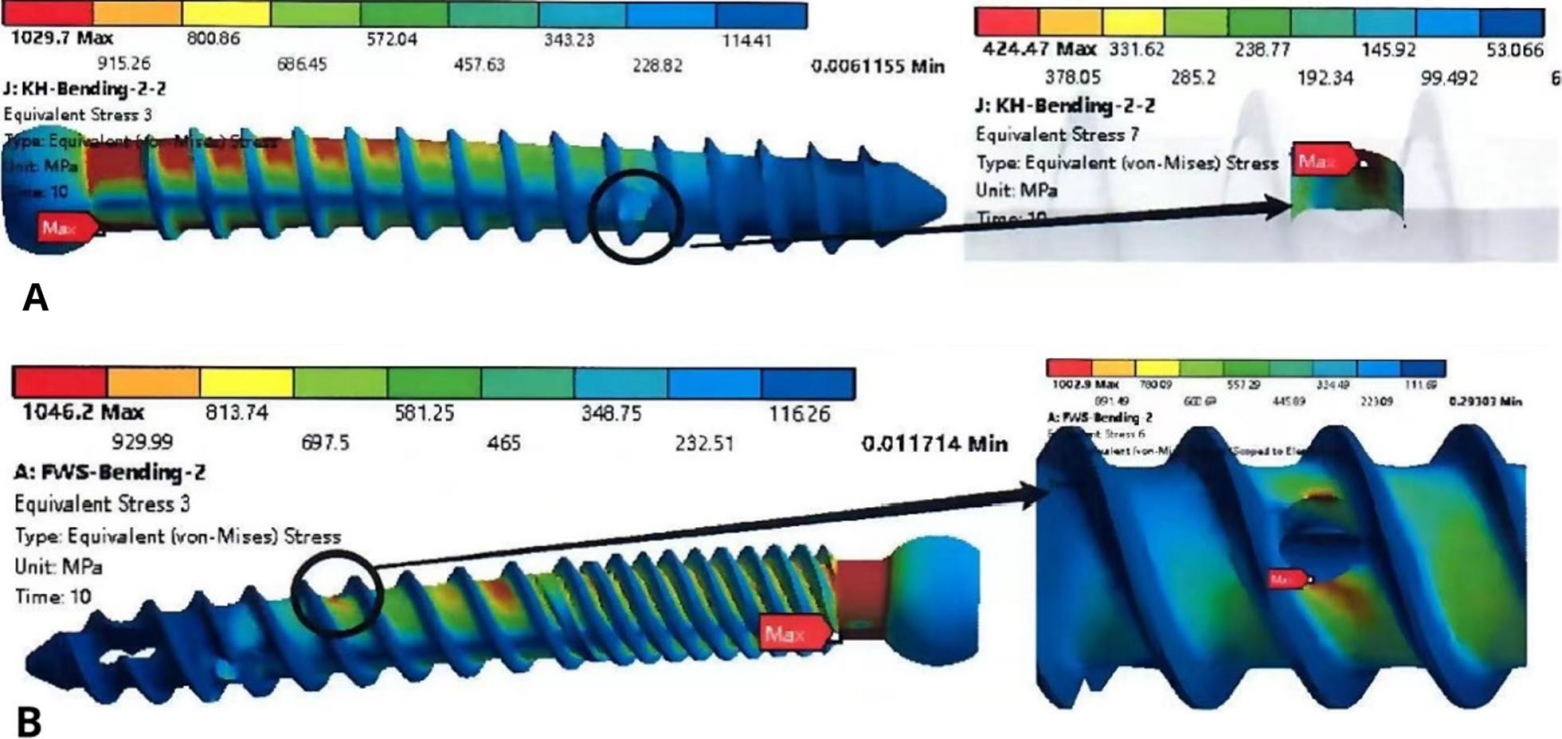
Stress cloud diagrams of first-generation (**A**) and second-generation (**B**) cement-injectable cannulated pedicle screws

The results of the simulated dynamic bending test (Fig. 12) show that the overall stress distribution of the second-generation CICPS was more uniform, and the stress distribution of the proximal side hole was equal to the maximum stress. The stress distribution of the first-generation CICPS was concentrated in the neck, and the stress on the proximal side hole was significantly lower than the maximum stress.

**Fig. 13:**
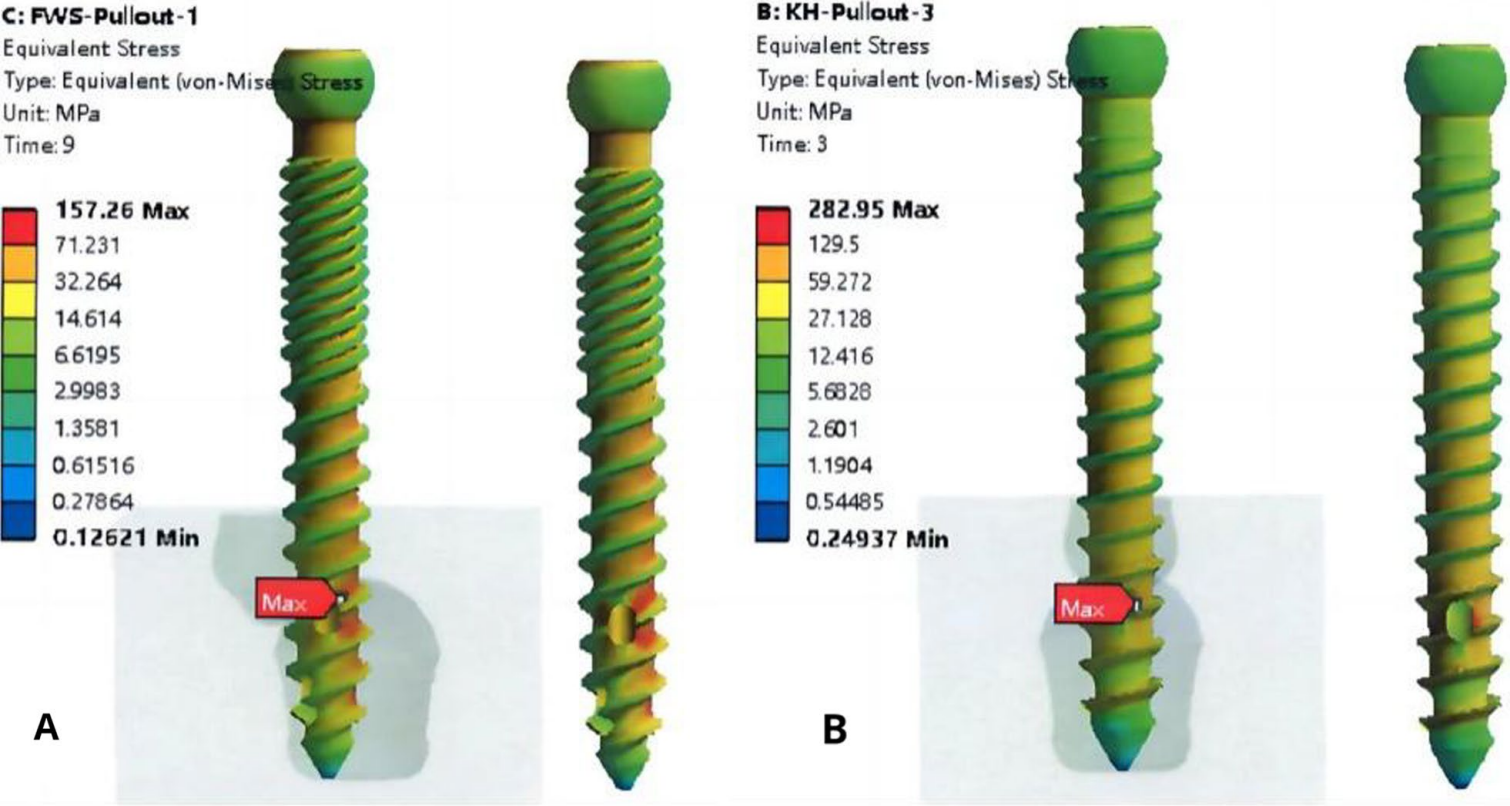
Stress cloud diagrams of second-generation (**A**) and first-generation (**B**) cement-injectable cannulated pedicle screws

The results of the simulated maximum axial pullout force test (Fig. 13) showed that under the same displacement, the screw–cement interface stress of the second-generation CICPS was smaller than that of the first-generation CICPS and less likely to fail.

## Discussion

Transpedicular screw instrumentation is regarded as a proven surgical procedure in the treatment of spinal diseases, achieving rigid stability in the anterior, middle, and posterior layers of the spine. However, surgical failure due to loosening, breaking, or pulling of the screw can occur, with screw pullout being one of the most common clinical complications [30, 31].

Many studies have reported methods to improve pedicle screw stability, including changing the shape of the screw, redesigning the screw threads, increasing the screw diameter, and reducing the screw pitch [12, 32–37]. Brasiliense et al. [38] used human vertebrae and PU foam blocks to compare screw insertion torque and maximum pulling force between conventional and double-threaded screws. Their results showed that the use of double-threaded screws significantly increased the insertion torque. Ramaswamy et al. [13] used PU at densities of 0.32, 0.24 and 0.16 g/cm^3^ to simulate normal bone, reduced bone mass, and osteoporotic bone, respectively, to compare screw fixation stability. They concluded that screw fixation strength significantly correlated with bone density and thread design. An osteoporosis-simulating PU bone block was used in our study. The second-generation screw adopts a double-thread design because of its smaller pitch and greater number of threads, thus, resulting in a larger contact area with the bone block, greater influence of friction at the screw–bone interface, and higher screw-in torque than that of the first-generation screw. A higher torque also means a stronger fixation effect, reducing the probability of screw pullout and further improving screw stability.

In the static bending experiment, the limit bending moment of the second-generation screw was lower than that of the first-generation screw, indicating that the first-generation screw should have better ductility. However, in the dynamic bending experiment, the force of the limit bending moment of the first-generation screw was less than that of the second-generation screw; in the maximum shear force experiment, the second-generation screw could withstand more cycles under the same force. Unlike conventional screw breakage that occurs in the neck, breakage of the second-generation screw occurs in the proximal lateral hole of the screw. We have reason to believe that the cause of this phenomenon is that the double-thread design changed the original stress distribution, making the stress distribution of the second-generation screw more uniform. Screw fracture occurred in the proximal side hole because it is closer to the thread change; structural changes are more susceptible to the influence of mechanics and are also more likely to exhibit morphological changes.

**Fig. 14:**
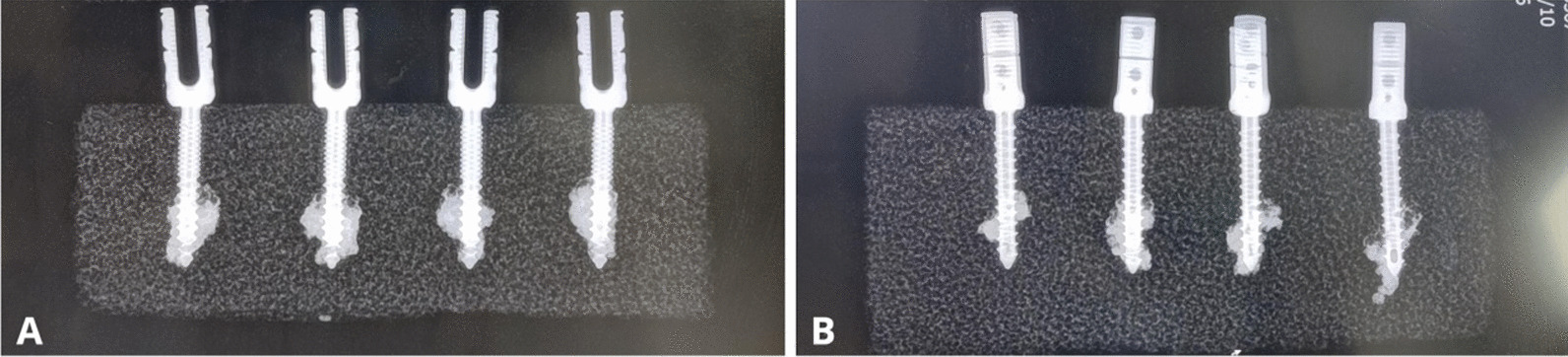
CT scans of PMMA distribution around the second-generation (**A**) and first-generation (**B**) cement-injectable cannulated pedicle screws

**Fig. 15:**
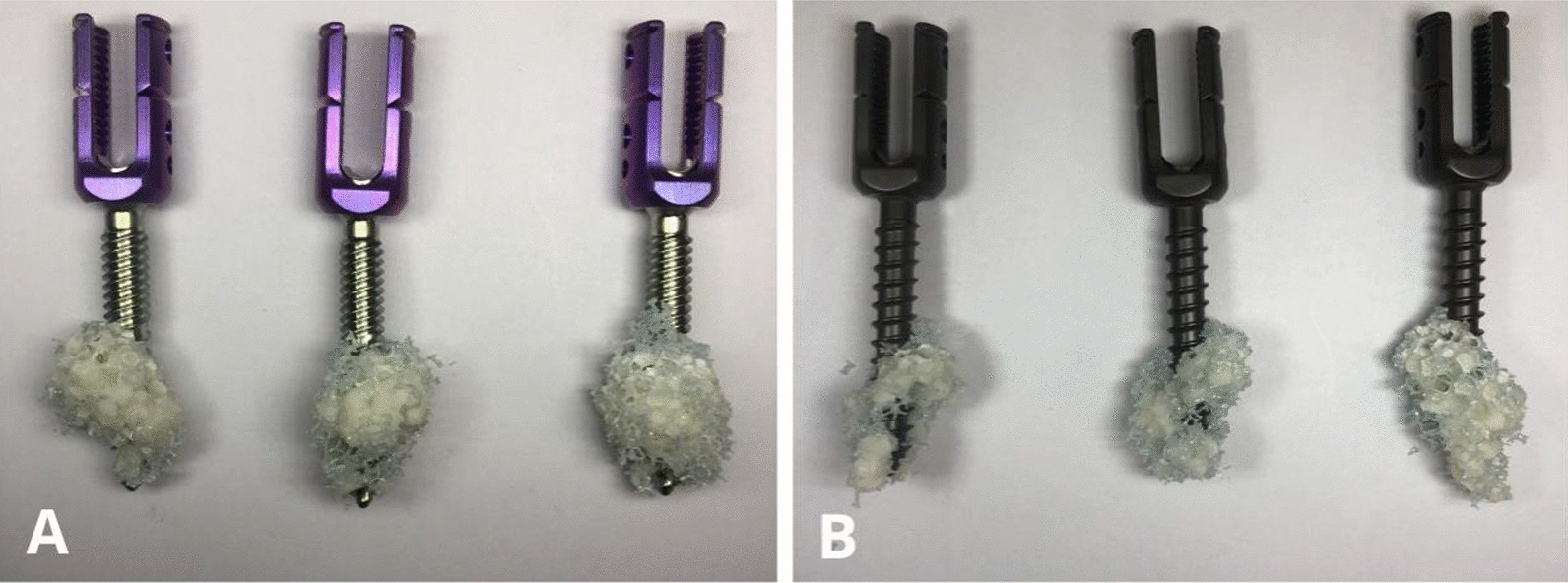
Removal from the bone block of second-generation (**A**) and first-generation (**B**) cement-injectable cannulated pedicle screws

Many studies have reported the use of PMMA to improve the stability of osteoporotic spinal pedicle screws [39–45]. Liu et al. [45] found a significant positive correlation between screw stability and PMMA volume. However, an excessive increase in PMMA volume does not significantly improve screw stability but rather increases the risk of cement leakage. Therefore, 1.5 mL is the optimal volume for PMMA injection to ensure the stability of the screw and safety of the cement. In the first-generation CICPS, the cement was concentrated on the side of the screw, which increased the leakage rate of the cement. Therefore, we improved the design such that the three side holes were evenly distributed on the surface of the screw, aiming to alter the distribution of the cement. In our study, the screws were assessed by X-ray imaging and CT after injection of 1.5 mL PMMA. PMMA showed a spherical distribution in the second-generation CICPS (Fig. 14A) and a conical distribution in the first-generation CICPS (Fig. 14B). In the second-generation CICPS, PMMA was more evenly distributed and broader along the screw axis. In the maximum pullout force experiment, the second-generation screw required a greater pullout force, indicating that under the same volume, the distribution form and scope of the cement can change the maximum pullout force of the screw, enabling the screw to achieve greater stability. After pullout, the cement was tightly connected to the screw, without loosening or cracking (Fig. 15).

In the finite element analysis, the simulated maximum shear force test showed that the neck maximum stress value of 1029.7 N for the first-generation screw was much higher than the maximum stress value of the proximal side hole at 424, 27 N, whereas the neck maximum stress value of 1046.2 N for the second-generation screw was not significantly different from the maximum stress value of the proximal side hole at 1002.9 N. This result is compatible with the mechanical experiments, which further shows that the double-thread design can change the stress distribution and failure form of the screws. The simulated maximum pullout force test of the screw–cement–bone interface as a whole showed that the force of second-generation CICPS (157.26 N) is less than the force of first-generation CICPS(282.95 N). The results show that more evenly distributed cement allows the screw–cement–bone interface to bear more uniform forces and results in greater stability between the internal structures.

### Limitations

First, simulated osteoporotic cancellous bone was selected for use in this study; however, the actual vertebral body is composed of cancellous bone and skin mass, which may result in some deviations from the experimental data. Second, the stress process was the result of a series of complex and comprehensive effects, and mechanical tests were completed under specific conditions and parameter sets; thus, the experimental results are not fully applicable to clinical practice. Finally, it is difficult to accurately simulate the real properties of materials using their geometric properties, which means that further experiments are required.

Future studies should collect more data in animal models of osteoporosis to further verify the safety and stability of second-generation CICPS prior to clinical application.

## Conclusions

The double-thread design results in a stronger pullout force, and the evenly arranged side holes make the cement package more uniform, resulting in less screw–cement–bone interface stress. The experimental results suggest that second-generation CICPS are safer and more stable than first-generation CICPS and represent a better choice for the surgical treatment of osteoporosis and spinal degenerative diseases.

## Data Availability

The datasets supporting the conclusions of this article are included within the article.

## Abbreviations

CICPS: Cement-injectable cannulated pedicle screws
CT: Computed tomography
PMMA: Polymethylmethacrylate
PU: Polyurethane
